# Theoretical study of the properties of X-ray diffraction moiré fringes. II. Illustration of angularly integrated moiré images

**DOI:** 10.1107/S2053273319004601

**Published:** 2019-06-26

**Authors:** Jun-ichi Yoshimura

**Affiliations:** a Sakai 5-13-2-A322, Musashino, Tokyo 180-0022, Japan

**Keywords:** diffraction moiré fringes, rotation moiré, *Pendellösung* oscillation, gap phase difference, integrated moiré images

## Abstract

Using a theory of X-ray diffraction moiré fringes developed in a previous paper, labelled Part I [Yoshimura (2015 ▸). *Acta Cryst*. A**71**, 368–381], the X-ray moiré images of a silicon bicrystal having a weak curvature strain and an interspacing gap, assumed to be integrated for an incident-wave angular width, are simulation-computed over a wide range of crystal thicknesses and incident-wave angular width, likely under practical experimental conditions. Along with the simulated moiré images, the graphs of characteristic quantities on the moiré images are presented for a full understanding of them. The treated moiré images are all of rotation moiré. Mo *K*α_1_ radiation and the 220 reflection were assumed in the simulation. The results of this simulation show that fringe patterns, which are significantly modified from simple straight fringes of rotation moiré, appear in some ranges of crystal thicknesses and incident-wave angular width, due to a combined effect of *Pendellösung* oscillation and an added phase difference from the interspacing gap, under the presence of a curvature strain. The moiré fringes which slope to the perpendicular direction to the diffraction vector in spite of the assumed condition of rotation moiré, and fringe patterns where low-contrast bands are produced with a sharp bend of fringes arising along the bands are examples of the modified fringe pattern. This simulation study provides a wide theoretical survey of the type of bicrystal moiré image produced under a particular condition.

## Abstract 2

Using a recently developed moiré-fringe theory of X-ray diffraction, the angularly integrated moiré images of a lightly strained silicon bicrystal having an interspacing gap were simulation-computed over a wide range of crystal thicknesses and incident-beam angular width.

## 1. Introduction   

In a previous paper (Yoshimura, 2015 ▸), hereafter referred to as Paper I, a theory of X-ray diffraction moiré fringes from a bicrystal specimen was given, and the properties of the moiré image derived by this theory were explained by showing examples of plane-wave moiré images computed by the theory. However, the practically observed moiré images are integrated images for the angular spread of the incident X-ray wave. Therefore, the illustration of plane-wave images alone is incomplete for the study of moiré images, although they are important as the basics in moiré-fringe study. By showing the simulation of integrated images, the theory would become better understood, thus making it useful for practical problems. Therefore, in this paper, similar to the previous presentation of plane-wave moiré images, a series of integrated moiré images from a bicrystal specimen having a weak curvature strain and an interspacing air gap are simulation-computed and surveyed according to this moiré-fringe theory.

A newly occurring problem in treating integrated images is the effect of gap phase difference (hereafter gap phase) which is involved in the total interference phase of the wavefield [see equations (8) and (9*b*)[Disp-formula fd9b] shown later]. When we treat plane-wave images, the gap phase does not become a significant problem, except when the front component crystal of the bicrystal (hereafter front crystal) is strained (see Fig. I-12; equations and figures in Paper I are indicated by the Roman numeral I attached to the equation or figure numbers). However, in integrated images the gap phase plays an essential role in the formation of the interference fringe pattern. As explained in Paper I (see Fig. I-2 and the succeeding equations), the gap phase is produced when the diffracted waves from the front crystal are propagated through the interspacing gap, and is added to the total phase difference of the wavefield. This gap phase varies with the glancing angle of the incident wave [see equation (9*b*)[Disp-formula fd9b] later], thereby causing a variation in the fringe position through a variation in the total phase difference of interference. By the integration of such small fringe-position variations, the resulting moiré-fringe pattern is significantly modified and the fringe contrast is decreased. To expand on the gap phase, its effect was first studied as gap interference fringes in X-ray topographs in theory and experiment (Authier *et al.*, 1968 ▸; Hart & Milne, 1970 ▸). The expression of the gap phase in plane-wave X-ray diffraction and a rocking-curve measurement from a gapped bicrystal of silicon have been reported by Yoshimura (1991 ▸).

## 2. Theoretical   

The integrated moiré images observed and discussed in this paper were computed as an integral of the plane-wave image intensity (diffracted-wave image) [see equation (I-20)] as follows:
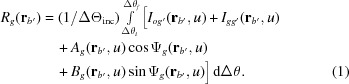



This integration may be understood to be a convolution integral of the plane-wave intensity function and a rectangular function of the peak height 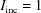 and angular width 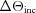 of the incident wave. The variable of integration 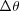 represents the deviation angle from the exact Bragg position when the X-ray wave is incident on the front crystal of the bicrystal. When we denote the upper and lower limit of integration by 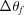 and 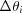, respectively, the angular width of the integration, 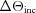, and the mid position of the integration width, 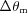, are given, respectively, as follows:
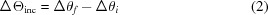


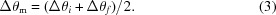



For the meaning and expressions of 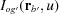, 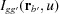, 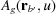 and 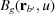 in the integrand in equation (1), see equations (I-22*a*,*b*) and (I-23*a*,*b*). [However, it must be stated here that the present expression of equation (I-22*a*) has an error: ‘
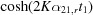
’ in the expression must be corrected to ‘
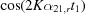
’. Although the error is present in the text, the computations in the paper were all made correctly using the correct expression.] In the calculation of the functions 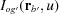 *etc*. above, the following three deviation parameters are relevant [see equations (I-45), (I-46*a*,*b*)]:
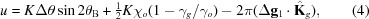


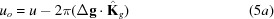


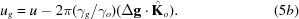



Here, *u* is the deviation parameter corresponding to the deviation angle 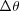 above; 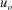 and 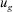 are the deviation parameters when the transmitted and diffracted waves (*O* and *G* waves, respectively) emerging from the front crystal are incident on the rear component crystal (hereafter rear crystal) to excite the transmitted and diffracted waves once more; *K* is the wavenumber in vacuum, and 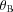 is the Bragg angle; 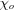 is the 0th Fourier component of the dielectric susceptibility of the crystal; 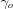 and 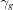 are the direction cosines of the transmitted and diffracted beams, respectively, with respect to the normal to the entrance surface of the front crystal; 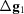 is a local change in the reciprocal-lattice vector from the 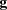 vector for the perfect region in the front crystal; 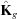 = 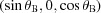 and 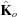 = 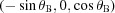 denote unit vectors along the direction of the diffracted and transmitted waves, respectively. The argument 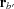 in the functions 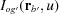 *etc*. is a vector referring to a position on the exit surface of the rear crystal. 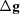 in equations (5*a* [Disp-formula fd5a]), (5*b*)[Disp-formula fd5b] is defined as
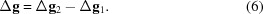


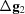
 is a change in the reciprocal-lattice vector in the rear crystal, relative to the reciprocal-lattice vector 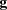 in the front crystal. This 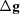 is the reciprocal-vector difference between the front and rear crystals, which is relevant in the production of moiré-fringe patterns. It is expressed as
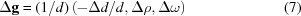
in the orthogonal coordinate system with the *yz* plane placed on the diffracting lattice plane (see Figs. I-2, I-3); 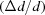 = 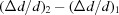, 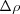 = 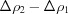, 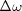 = 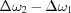; 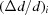, 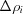 and 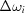 (*i* = 1, 2) denote, respectively, the lattice-spacing difference, and the rotation of the lattice plane about the *z* and *y* axes in the front crystal (*i* = 1) and rear crystal (*i* = 2), relative to an unstrained region in the front crystal; *d* is the lattice spacing.

When we assume symmetric Laue geometry where the diffracting lattice plane is perpendicular to the crystal surfaces, the term representing the phase difference of the interference in the integrand function in equation (1) is expressed as
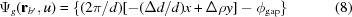
[see equations (I-34)–(I-37) for details]. Here, 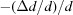 and 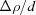 in the first term on the right side are the *x* and *y* components, respectively, of 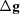, as shown in equation (7); the moiré-fringe pattern is intrinsically drawn by these two quantities. The second term represents the gap phase as mentioned in Section 1, and is given by [see equations (I-11), (I-21) and (I-34)]:
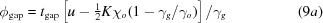


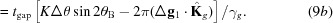



Here, 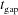 is the width of the gap. If the gap is filled with some high-density material such as silicon oxide, instead of air, an added phase owing to the passage through it should be taken into account. Then, the gap phase above is modified to
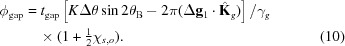
Here, 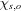 is the Fourier component of the dielectric susceptibility of the filled material. However, while 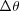 remains a minute quantity, this correction is an even more minute quantity. Therefore, the correction is considered to be practically unnecessary.

The preparations for computing moiré images are ready now. However, to discuss the characteristics of the interference patterns of the computed moiré images, we also need the wavefield expression in which the two phase terms in equation (1) are arranged to form one term. For this reason, we prepare the following expression by expanding and rearranging equations (1) and (8):
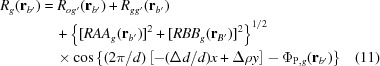
with
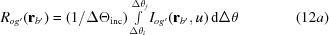


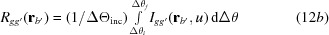


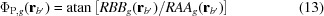


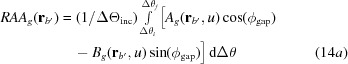


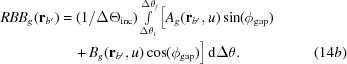



As shown in the expression in equation (11), 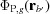 in equation (13) represents the contribution of *Pendellösung* oscillation (hereafter PL oscillation) to the total interference phase of the wavefield. In this sense it may be called the *Pendellösung*-connected term (hereafter the PL phase), although it is also connected with the gap phase as seen from equations (13) and (14*a*)[Disp-formula fd14a], (14*b*)[Disp-formula fd14b]. In combination with the effect of the gap phase, the phase 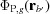 becomes a source of oscillations that are more complicated than the simple PL oscillation. From the expression in equation (11), the fringe contrast in moiré images is given as follows:
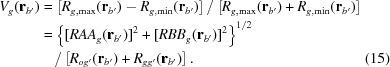



As to the numerical conditions for computation, the Si 220 reflection with Mo *K*α_1_ radiation (0.70926 Å) was assumed throughout this paper; 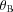 = 10.64°. The linear absorption coefficient is μ = 1.462 mm^−1^. [All these values are taken from Pinsker (1978 ▸).] As already mentioned above, the diffracting (220) lattice plane was assumed to lie perpendicular to the crystal surfaces in the symmetric Laue geometry (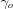 = 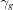). The crystal surfaces in the bicrystal specimen are all parallel to one another (see Fig. I-2). Except for the case of Fig. 7, the differences in the spacing and orientation of the lattice plane from the reference region were assumed as follows: the front crystal is strain free, namely 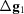 = 0 [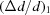 = 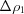 = 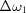 = 0] and 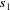 = 0 [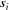 (*i* = 1, 2) denotes the strength of the curvature deformation of the crystals around the *y* axis]; the rear crystal, on the other hand, has a non-zero reciprocal-vector difference 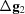 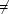 0, *i.e.* 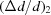 = 0, but 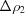 = *d*/0.045 (rad); in addition, the crystal has a curvature deformation of *s*_2_ = 0.045′′ mm^−1^ (radius of curvature *r* = 4600 m) and thereby a rotation of the lattice plane around the *y* axis 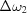 = 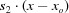 is induced over the whole crystal. Here, 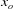 denotes a position on the crystal surface corresponding to the centre of curvature, and was assumed to be 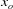 = 9.0 (mm). The deviation parameters in equations (4) and (5*a*)[Disp-formula fd5a], (5*b*)[Disp-formula fd5b] become
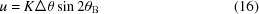


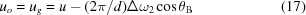
based on the assumptions above. All these conditions are the same as those in the computation of the plane-wave moiré images in Paper I. A rotation-moiré pattern should appear in the diffracted images, similar to the case of the previous plane-wave images. Hereafter, the thicknesses of the component crystals, the gap width, and the *x* position in the crystals and moiré images are given in units of mm (the *y* position is not referred to in this paper). The computation of moiré images was made with *Visual Basic.NET* version 2003, and the intensity and contrast in the output images were adjusted in image processing so as to be best suited for observation. Computation of the graphs of the characteristic quantities 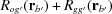, 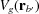 *etc*. was made using *SigmaPlot* version 11.0.

## 3. Results and discussion   

The commonly assumed numerical conditions for the computation were as stated above. In the following we observe and discuss the moiré images obtained by changing the thicknesses of the component crystals 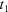 and 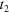 of the front and rear crystals, respectively, and the incident-wave angular width 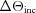. The gap width 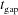 was also set at different values in the computation.

### 3.1. Moiré images of bicrystals of *t*_1_ = *t*_2_ = 0.8 (μ*t*_1,2_ = 1.170)   

#### 3.1.1. Images with *t*_gap_ = 0   

**Figure 1:**
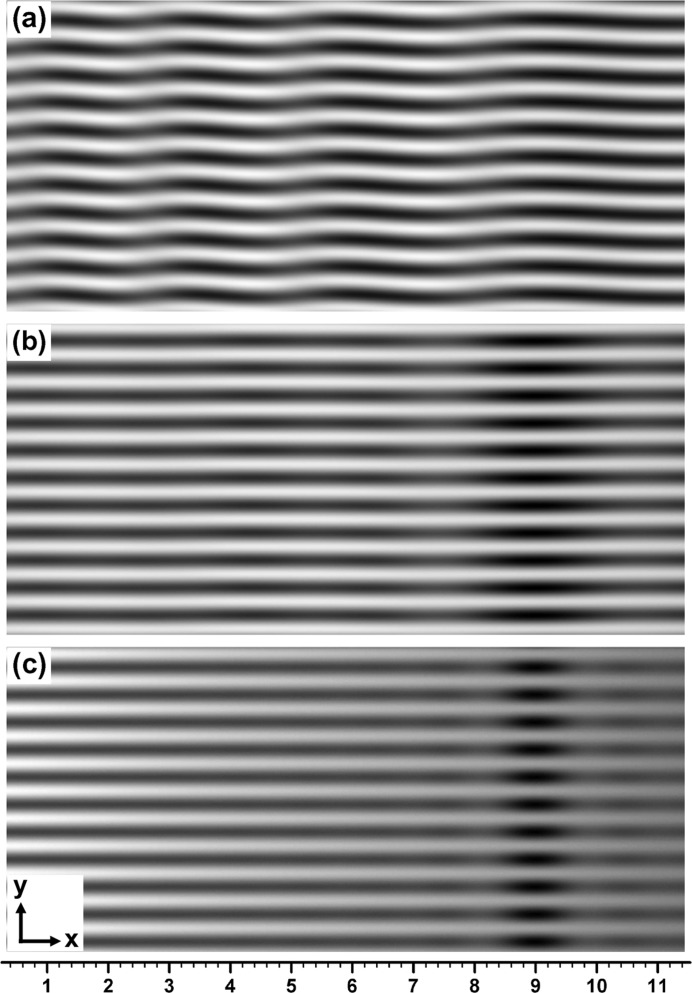
Computer-simulated integrated moiré images of X-ray diffraction for a silicon bicrystal specimen with component-crystal thicknesses of 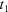 = 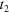 = 0.8 mm and gap width of 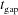 = 0. The 220 reflection with Mo *K*α_1_ radiation was assumed. Angular widths of the incident X-rays were set to be 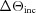 = 0.04′′, 0.50′′ and 10.0′′ for images (*a*), (*b*) and (*c*), respectively. The mid deviation angle was set to be 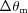 = 0.32′′. The graduations at the bottom of the image (*c*) are given in mm. For further information see text.

First, we show some integrated moiré images when 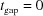 in Figs. 1 ▸(*a*)–1 ▸(*c*). These are representative of images obtained when 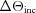 is increased gradually, starting from a minute value. To compare unambiguously with previous plane-wave images, the mid deviation angle in the integration was set to be 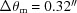 for which angle the plane-wave images Fig. I-8 *etc*. were computed; it is slightly off the peak position of the diffracted-intensity curve. In accordance with the assumption of 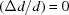 and 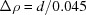, moiré fringes with a fringe spacing Λ = 0.45 mm in the *y* direction are produced. Needless to say, these images are topographs of the diffracted wave (*G* image). The images are shown in the way that they are viewed from the emerging-beam side, and the *x*- and *y*-coordinate axes are taken as shown in Fig. 1 ▸(*c*). The image contrast is reproduced in such a way that white contrast indicates higher intensity, which is the opposite to convention. The diffraction vector 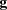, which is not shown in the images, is directed from the left to the right along the *x* axis (see *e.g*. Figs. I-1 and I-3).

**Figure 2:**
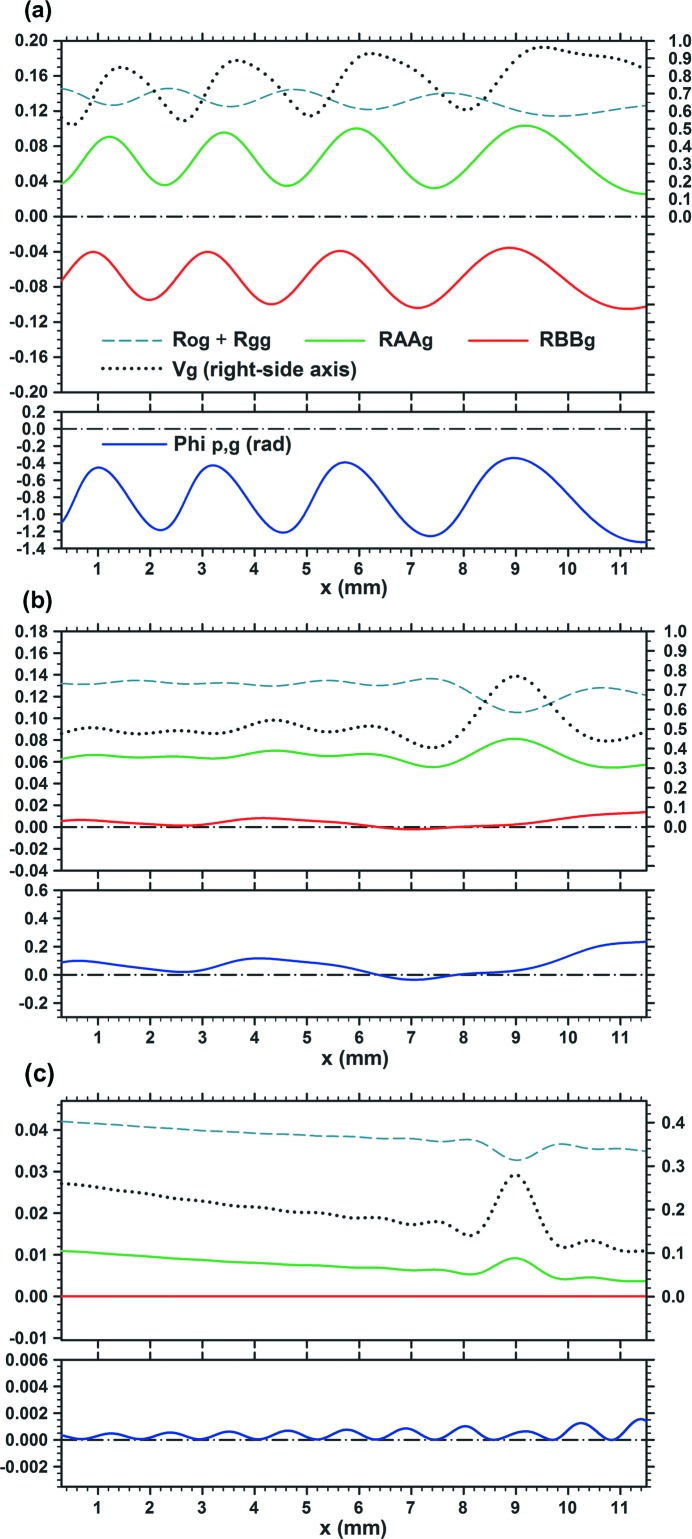
Curves of characteristic quantities of the moiré images in Fig. 1 ▸, showing local variations of the mean image intensity [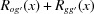], fringe contrast 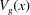, amplitude intensities 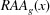 and 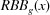, and PL phase 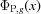. Graphs (*a*), (*b*) and (*c*) are related to the moiré images (*a*), (*b*) and (*c*), respectively, in Fig. 1 ▸. For further information see text.

Fig. 1 ▸(*a*) shows the image computed with angular width 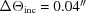, which is very close to the plane-wave condition. In fact, this moiré image is very similar to the plane-wave image in Fig. I-8(*a*). When 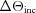 was increased, the integrated image remained nearly the same as the plane-wave image up to about 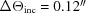, and a somewhat clear difference between the integrated and plane-wave images began to be found from about 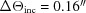. When 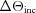 was increased further, an oscillation in the fringe lines and a change in the image intensity distribution with 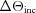 continued to be observed up to about 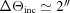, although the oscillation was of very small amplitude and the intensity distribution change was very gentle. Fig. 1 ▸(*b*) shows an example of a moiré image in such a middle region of 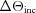 before reaching 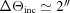. The fringe lines still show a slight oscillation in this image [see Fig. 2 ▸(*b*) later]. With the angular width of 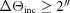, the fringe pattern had almost settled to the one shown in Fig. 1 ▸(*c*). Even when 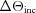 was increased further, the fringe pattern did not change significantly from the pattern in Fig. 1 ▸(*c*), although the image intensity and fringe contrast changed to some extent. It was confirmed by the present computation that such almost-settled fringe patterns continue to be observed up to 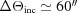, which may be referred to as the condition in Lang topography.

Fig. 2 ▸ shows the curves of the mean image intensity 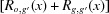 unrelated to the moiré interference, fringe contrast 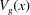, amplitude intensities 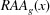 and 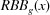 in the interference terms [in equation (1)], and of the PL phase 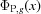 for the moiré images in Figs. 1 ▸(*a*)–1 ▸(*c*). These characteristic quantities were computed separately from the computation of moiré images. (From here on, the position variable in these functions is denoted by *x* instead of 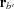 as in Section 2.) The results of the computation of the moiré images and characteristic curves agree well with each other. We can understand the characteristics of the moiré images clearly and in detail from these characteristic curves. Firstly, it can be pointed out that the phase curve 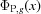 in Fig. 2 ▸(*a*) is very similar to the phase curve [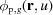] of the plane-wave image shown in Fig. I-9(*b*); it is seen that the wave forms of oscillation in these phase curves are both slightly asymmetric and lean to the left side. This similarity between the two phase curves confirms the correctness of the similarity mentioned above for the integrated and plane-wave moiré images [Fig. 1 ▸(*a*) and Fig. I-8(*a*)]. The fringe-line equation obtained by setting the argument of the cosine function in equation (11) to be equal to 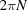 is given in this case (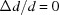) by
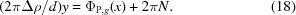
As seen from this equation, the shape of the fringe lines is governed by the phase curve 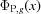.

It can be seen in Figs. 2 ▸(*a*)–2 ▸(*c*) that the oscillation of moiré-fringe lines is damped rapidly with an increase in the angular width 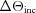, and the shape of the fringe lines approaches that of a horizontal straight line. This damping is caused by the convergence of 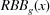 to the zero value, which is set as the numerator in the expression of 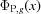 [equation (13)], while the denominator 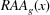 remains at a finite value. Although the curve of 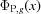 in the lower graph in Fig. 2 ▸(*c*) is drawn on an enlarged scale in the vertical direction, to show that an infinitesimal oscillation still exists, it is practically a horizontal straight line in the scale of Fig. 2 ▸(*a*) or 2 ▸(*b*). It must be added that the property 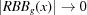 with increase in 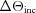 is true only when 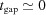; in the case of 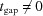, 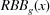 does not show such convergence. As seen from equations (14*a*)[Disp-formula fd14a], (14*b*)[Disp-formula fd14b], when 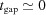,
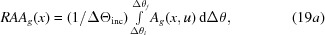


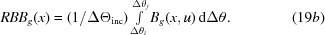



In addition to the fringe lines, the fringe contrast 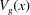 also shows an oscillatory variation, and its minimum value becomes zero in some cases. Such oscillatory variations in 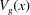 can generally be understood to be governed by the oscillations in 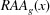 and 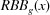. As seen from equation (15), the variation in 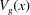 is approximately estimated by
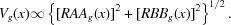



A shallow drop in the curves of the mean image intensity 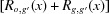 around 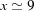, seen in the upper graphs in Figs. 2 ▸(*b*), 2 ▸(*c*), is considered to show a secondary extinction effect in the crystal diffraction. In the images [Figs. 1 ▸(*b*), 1 ▸(*c*)] this reduction in intensity appears as a black, diffuse band image. The rear crystal becomes 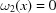 in this position 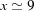, and the front and rear crystals become locally parallel to each other. Similar reductions in intensity are also observed with the crystal thicknesses of 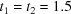 and 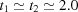 (see Figs. 5 and 8, respectively), but disappear with 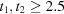 (see Fig. 9). When both the front and rear crystals were unbent, *i.e.* 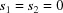, and 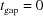, but 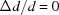 and 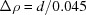 as in the other cases described above, the moiré images were properly of horizontal straight fringes (hereafter HS fringes) with any value of 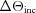, since all the quantities concerned become independent of the *x* coordinate. Then, while the mean image intensity 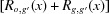 is considerably decreased compared with the images in Fig. 1 ▸, fringe contrast 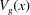 is greatly increased (see Figs. 11 and 12 shown later).

#### 3.1.2. Images with *t*_gap_ = 0.05   

When the supposed bicrystal specimen comes to have a gap of finite width, some change from images with 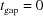 is expected to appear. However, up to about 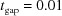, the obtained moiré images were almost unchanged from the ones with 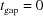, by comparison of the images (not the characteristic curves). Even when the gap width became 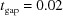, there was almost no change from the image with 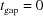, although a small difference came to be found with a large value of 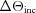. Though not clearly recognized in the images, the oscillation in the curve of 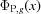 appeared to increase compared with the level when 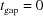, and the fringes sloped slightly upwards to the right. The previous moiré images by Brádler & Lang (1968 ▸) and by Lang (1968 ▸) are considered to be taken under such a condition, but with no curvature strain in the specimens.

**Figure 3:**
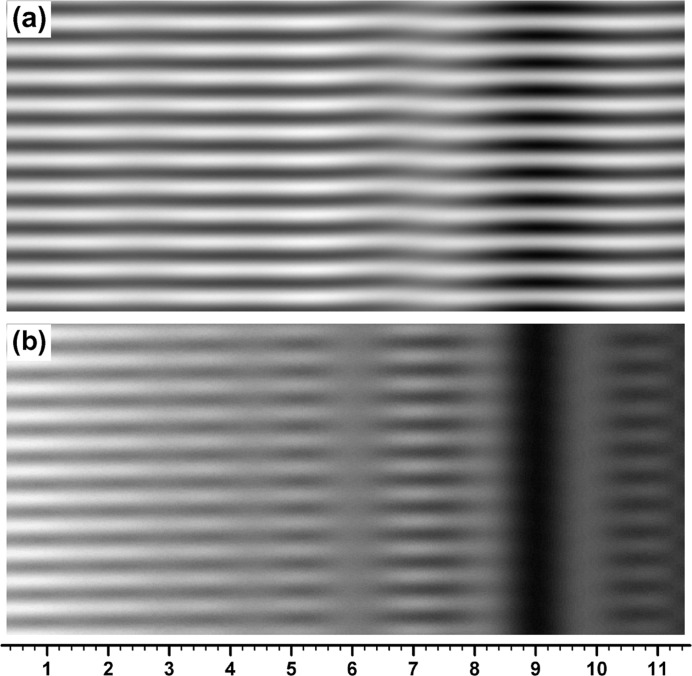
Moiré images simulation-computed under similar conditions to those for Fig. 1 ▸, except for the gap width of 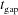 = 0.05. 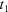 = 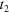 = 0.8. (*a*) Moiré image with 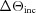 = 0.50′′, (*b*) moiré image with 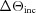 = 10.0′′. For further information see text.

**Figure 4:**
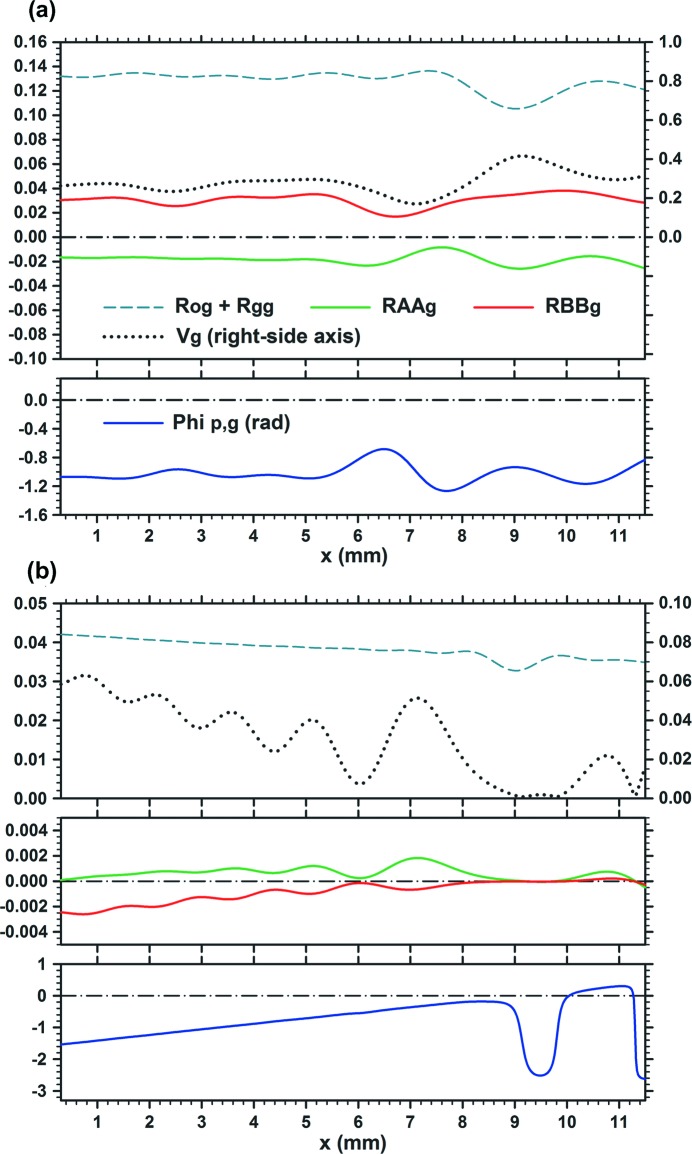
Characteristic curves of the moiré images in Fig. 3 ▸. Graphs (*a*) and (*b*) are related to the moiré images (*a*) and (*b*), respectively, in Fig. 3 ▸.

When the gap width was increased further, the difference from images with 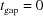 gradually became clear. Moiré images with 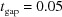 and graphs of the characteristic curves concerned are shown in Figs. 3 ▸ and 4 ▸, respectively. Figs. 3 ▸(*a*), 3 ▸(*b*) are to be compared with Figs. 1 ▸(*b*), 1 ▸(*c*), respectively. When the angular width 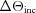 remains small (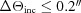), images with 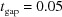 are nearly the same as those with 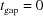. However, when the angular width was increased further, the difference in images began to be noticeable. Though not clear in the images, the graphs in Fig. 4 ▸ show that the fringe contrast 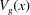 is lowered considerably with 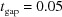, compared with the case with 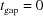. When the angular width was increased up to 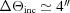, the fringe pattern had almost settled to the one shown in Fig. 3 ▸(*c*), and remained almost unchanged with further increase in 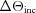.

### 3.2. Moiré images of bicrystals with *t*_1_ = *t*_2_ = 0.8 (μ*t*_1,2_ = 1.170) and *t*_gap_ = 0.24   

#### 3.2.1. General survey of resulting images   

**Figure 5:**
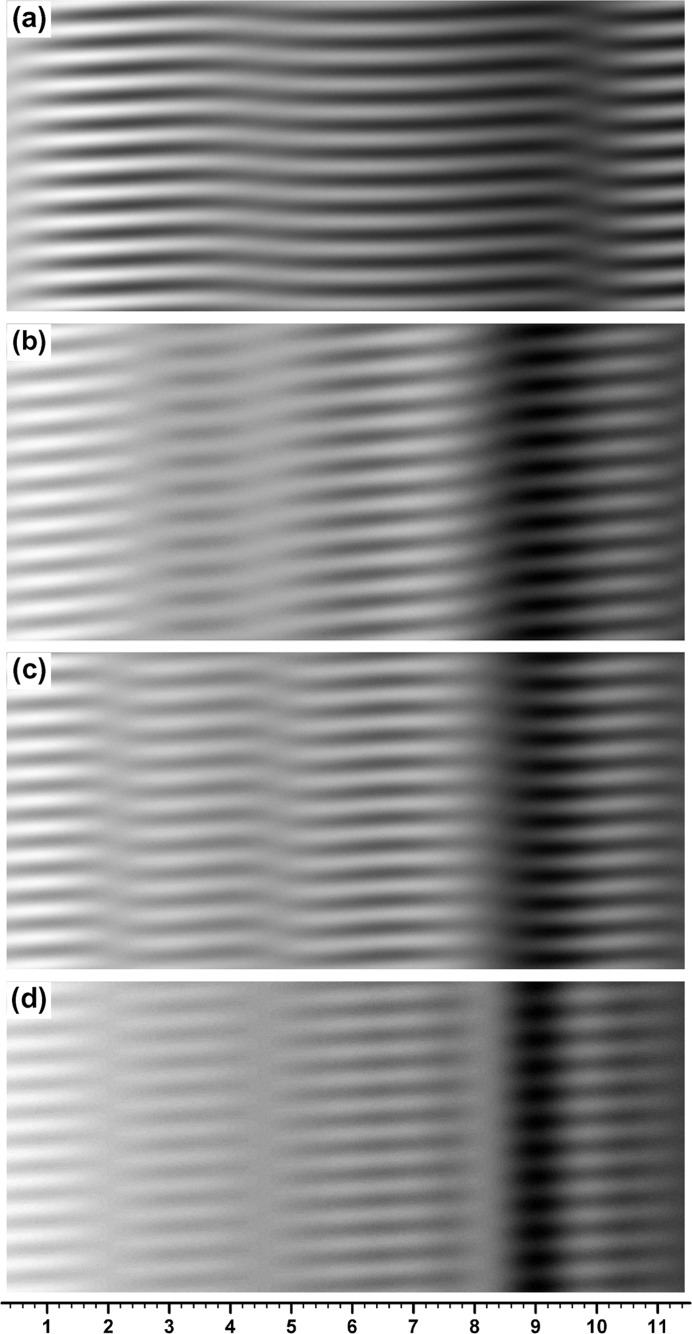
Moiré images computed under similar conditions to those in Fig. 1 ▸, except for the gap width of 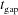 = 0.24 and the mid deviation angle of 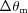 = −0.12′′. 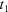 = 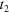 = 0.8. The incident-wave angular widths for the computed moiré images were assumed to be: for the image (*a*) 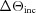 = 0.40′′, (*b*) 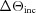 = 1.08′′, (*c*) 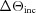 = 1.20′′ and (*d*) 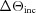 = 10.0′′. For further information see text.

Figs. 5 ▸(*a*)–5 ▸(*d*) show some representative moiré images when the crystal thicknesses were 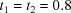, but the gap width was set to be 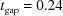. Here, computation was performed with the mid deviation angle 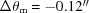, which was changed from 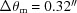 for the images in Figs. 1 ▸ and 3 ▸. In Paper I, the plane-wave image in Fig. I-10(*a*) was computed under this condition, namely 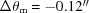. When the angular width 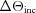 was increased gradually, the integrated images did not show any obvious difference from the plane-wave images up to about 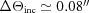; however, sharp differences became visible from about 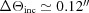. Fig. 5 ▸(*a*) shows an example of such an integrated image with a relatively small 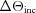. For the increased gap width of 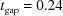, the fringe patterns became considerably modified from the simple HS fringes. Representative examples of such moiré images when 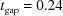 and 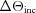 is of middle magnitudes are shown in Figs. 5 ▸(*b*) and 5 ▸(*c*); they will be explained in detail later. In the case of 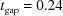 also, a large change in the fringe pattern was not observed with angular width larger than 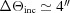, and the fringe pattern had almost settled to the one shown in Fig. 5 ▸(*d*). This image is roughly similar to the one in Fig. 3 ▸(*b*), but the slope of the fringes becomes larger than in Fig. 3 ▸(*b*) and vertical streaks or bands appear more strongly. The slope angle of the fringes was 3.5° in the image shown in Fig. 5 ▸(*d*), which was measured directly on the image, while it was 1.3° in the image shown in Fig. 3 ▸(*b*). While the slope angle appears to be related to the magnitude of 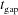, it is also thought to be related to the curvature strain in the crystal. To confirm this thought, computations of moiré images were made to confirm that the slope angle of the fringes is zero with 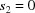, and increases with the value of 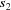.

#### 3.2.2. Effect of gap phase on the formation of fringe pattern   

In the following we discuss the images in Figs. 5 ▸(*b*) and 5 ▸(*c*) in detail. In the moiré image in Fig. 5 ▸(*b*) fringes slope by about 8° from the *x* axis, in the direct measurement on the image. Such a fringe pattern appears to be extraordinary for that of rotation-moiré fringes with 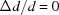. Although it is difficult to give a satisfactory explanation for this strange fringe pattern at present, the following is a partial explanation based on an incomplete study. As mentioned in Section 1, when the deviation angle 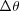 changes, the position of the fringe lines also changes due to the change in the gap phase 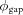 [see equations (8) and (9*b*)[Disp-formula fd9b]]. For a change of 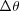 in the deviation angle, the gap phase changes by
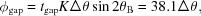
with 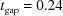 (here, 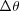 is given in arcsec). The fringe positions move upwards or downwards due to this phase change. Therefore, the effective width of the *N*th fringe is expanded by such fringe-position movements, and it becomes 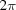 due to the 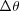 change in the overall width of 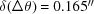, and 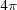 for 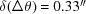 with its expansion forefronts reaching the central positions of the neighbouring (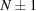)-th fringes. With the incident-wave angular width of 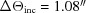 for the image under consideration, the range of one fringe extends to the distance of 3.3 times the fringe spacing to each side of its central position. Each fringe is thus expanded, and overlaps with neighbouring fringes. The patterns of obliquely extending fringes (hereafter OE fringes or OE fringe pattern) and of broadly horizontal fringes (BH fringes or BH fringe pattern), which are shown, respectively, in Figs. 5 ▸(*b*) and 5 ▸(*c*), are considered to be produced in connection with such a complicated fringe arrangement.

While simulating the formation process of the OE and BH fringe patterns, it was seen that the resulting fringe-line configuration in the integrated image is formed by connecting the regions where plane-wave fringe lines are most densely overlapped. Then, due to a slight difference in the overlap of plane-wave fringe lines and/or a slight change in the shape of the fringe lines, the resulting fringe pattern seems to be divided into OE and BH fringe patterns. While some fringes (in integrated images) continue to lie in their initial *y* positions, though making an oscillation with the *x* position, the other fringes switch their initial vertical positions so that they are connected to a neighbouring fringe at a site that is one spacing higher or lower, in the way of the continuation along the *x* axis. The fringes repeat such switching of the vertical position, thereby extending obliquely to a higher or lower position. Such a difference in the continuation behaviour of fringes seems to cause the difference of the OE and BH fringe patterns. However, further investigation of these fringe patterns is not an easy task, because it requires precise simulation of complicated fringe arrangements.

#### 3.2.3. OE and BH fringe patterns viewed in the related Φ_P,*g*_(*x*) phase curves   

**Figure 6:**
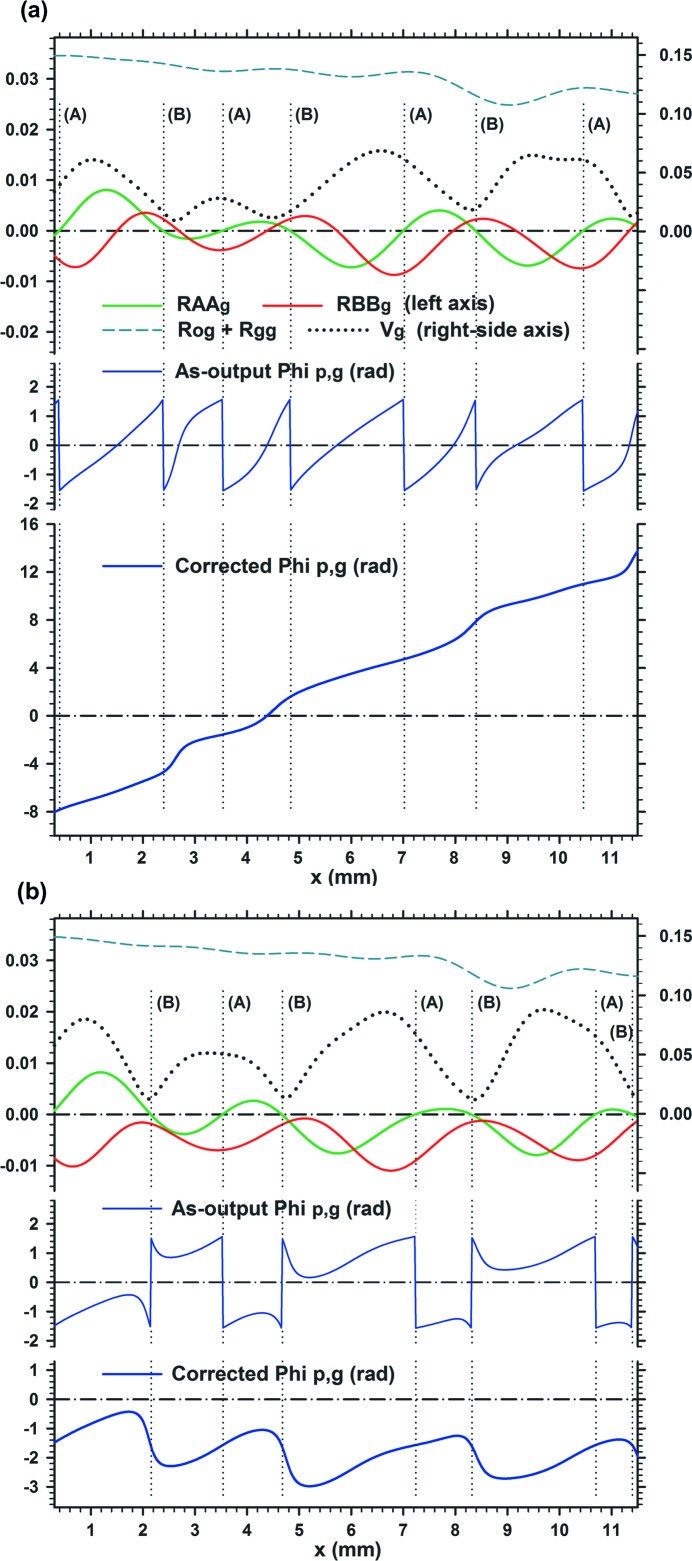
Characteristic curves of the moiré images in Fig. 5 ▸. Graph (*a*) is related to the moiré image (*b*) in Fig. 5 ▸, and graph (*b*) is related to the moiré image (*c*) in Fig. 5 ▸. For further information see text.

The formation of the OE and BH fringe patterns discussed above can be understood to a fair extent from the observation of 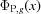 phase curves shown in Figs. 6 ▸(*a*), 6 ▸(*b*). In each of these figures, the ‘as-output 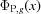
’ phase curve is additionally given in the middle-column graph to provide a good understanding of the ‘corrected 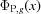
’ phase curve shown in the lowest graph. The as-output 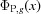 curves are phase curves computed using equation (13). They have discontinuous jumps of 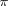 or 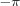, wherever 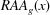 in the denominator in equation (13) crosses the zero line. These discontinuities were all corrected so that the as-output 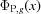 curves are changed to continuous curves of the corrected 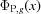 phase, as described in Fig. I-7. The corrected 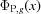 curve is the true phase curve, and it gives the shape of the fringe lines in the moiré image, as shown in equation (18). The moiré images are computed using equation (1), so that they conform with the corrected 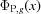 phase curve from the beginning.

It is seen in Fig. 6 ▸(*a*) for the OE fringes that the discontinuous phase change is 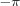 (seen from left to right) at all phase jumps in the as-output 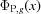 curve, and the curve position is raised by 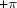 at every correction site. In more detail, there occur two types of phase jumps, *A* and *B*. While in the type-*A* jump the curves on both sides of the jumping site are connected to each other smoothly after the correction, in the type-*B* jump an upward bend remains in the corrected curve, to enhance the rise of the curve position. Such a rise in the vertical position of the phase curve is presumed to agree with the fringe-position rise of OE fringes described qualitatively in Section 3.2.2. In contrast to the case shown in Fig. 6 ▸(*a*), discontinuous phase changes of 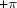 and 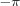 occur alternately in the as-output 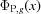 curve shown in Fig. 6 ▸(*b*) for the BH fringes. Although an upward slope of the phase curve is also seen in this case [see the corrected 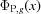 curve in the lowest graph], gradual rises in the curve position are cancelled by sharp downward bends at the type-*B* correction sites, to keep the phase curve broadly horizontal. If we want to inquire further into the behaviours of 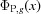 phase curves in these two cases, the study of the curves of 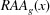 and 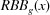 is necessary. However, it is omitted. So far, we have described the OE and BH fringe patterns of moiré images as representatives in the middle-magnitude domain of 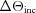. Roughly speaking, these two types of fringe patterns appear alternately with an increase in 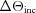 in the range from about 0.4′′ to about 4′′. The type of fringe patterns produced changes with 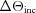. The frequency of occurrence of the two types seems to depend on the crystal thicknesses 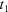 and 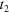. Images where the two types of fringe patterns co-appear in such a way that one type of fringe pattern is connected smoothly to another type also appeared fairly frequently.

#### 3.2.4. Low-contrast bands in the BH fringe pattern   

Further comments are made below on the BH fringe pattern in Figs. 5 ▸(*c*) and 6 ▸(*b*). At the positions of the type-*B* phase jumps in Fig. 6 ▸(*b*), 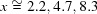, the fringe contrast 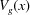 locally falls to produce a streak or band pattern, accompanying a sharp bend in the fringe lines. Although similar low-contrast bands can be seen in Figs. 5 ▸(*b*) and 6 ▸(*a*) for the OE fringe pattern, the fringe bends are not so sharp in that case. As seen in the graph in Fig. 6 ▸(*b*), sharp fringe bends occur where 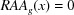 and 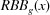 approaches its maximum. Then, 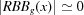, and the fringe contrast 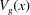 is minimized according to equation (15). The graphs of the as-output and corrected 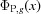 curves are similar to those of the phase curves in Fig. I-9(*a*) for the plane-wave image with which ‘abrupt fringe jump’ is explained, although the wave form in the phase-curve oscillation leans to the opposite side to the lean of the wave form in the present phase curve in Fig. 6 ▸(*b*). The curves of 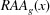, 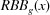 and 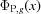 in Fig. 6 ▸(*b*) correspond to the curves of 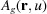, 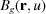 and 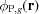 in Figs. I-9(*a*), respectively. On the basis of this similarity, it is evident that the ‘low-contrast bands’ in this paper are of the same origin as the ‘vertical bands of abrupt fringe jump’ for the plane-wave image in Paper I.

The band patterns mentioned above are produced as a result of oscillation with intensities 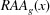 and 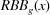, which were originally caused by the PL oscillation in the crystals. The contour or fringe patterns caused by the PL oscillation in the diffracted image of a curved crystal are called bend-extinction or equal-inclination fringes (Hirsch *et al.*, 1965 ▸; Sugii *et al.*, 1971 ▸). It is certain that the band pattern in the present moiré images is of the same kind as this PL oscillation-connected fringe pattern. From the computations made so far, it is confirmed that the number of bands produced is increased or decreased depending on the curvature strength 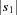 or 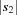. However, in the study for the case of 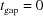, which is described in Section 1, it is observed that band patterns disappear in moiré images with 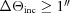, certainly due to smoothing of the intensity oscillation. Nevertheless, the band pattern in the moiré image in Fig. 5 ▸(*c*) is clearly observed even with an angular width of 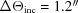. This disagreement is considered to indicate that the band pattern in the integrated moiré images with 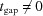 is produced not only by the PL oscillation, but also by a collaboration of the PL oscillation and gap phase effect. In this respect the band pattern under discussion is not entirely similar to the intrinsic equal-inclination fringes. It is tentatively referred to as the low-contrast band (LC band).

#### 3.2.5. Other remarks   

One more comment that should be made on Figs. 5 ▸(*c*) and 6 ▸(*b*) is regarding the direction of lean of the oscillation wave form in the fringe lines or the 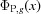 phase curve. In the phase curve in the plane-wave moiré image, the oscillation wave form always leans to the left side, as seen in Figs. I-8(*a*), (*b*) and Figs. I-9(*a*), (*b*). In the integrated images the oscillation wave form in question also leans to the left side as long as the angular width 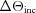 remains very small. An example of such a phase-curve oscillation wave form is seen in Fig. 2 ▸(*a*), in the case of 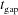 = 0. This characteristic of the leaning direction also holds in the case of 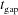 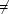 0. However, as the value of 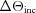 increases, the oscillation wave form leans to the right side, as seen in Fig. 4 ▸(*a*) and Fig. 6 ▸(*b*), via a neutral symmetric form in a narrow range of 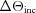. As studied by the computation of moiré images and characteristic phase curves, the oscillation wave form in the integrated images when 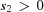 seems to generally lean to the right side except when 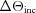 is very small. The reason for this characteristic is yet unknown.

**Figure 7:**
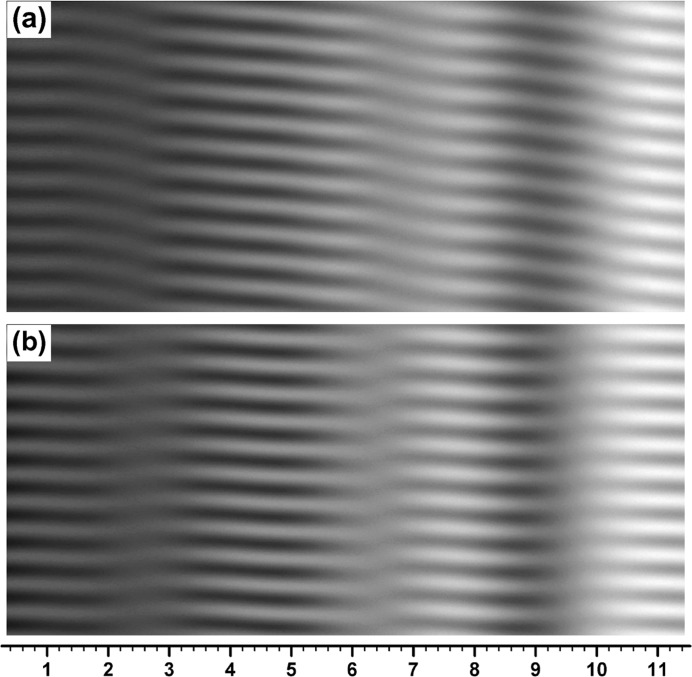
Moiré images computed under similar conditions to those of the two images in Figs. 5 ▸(*b*) and 5 ▸(*c*), but with the curvature deformation of the rear crystal reversed in sign, *i.e.* with *s*_2_ = −0.045′′ mm^−1^. The image (*a*) computed with 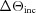 = 1.04′′ in this figure is compared with the image in Fig. 5 ▸(*b*), and the image (*b*) with 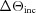 = 1.20′′ is compared with the image in Fig. 5 ▸(*c*).

With reference to the images in Figs. 5 ▸(*b*), 5 ▸(*c*), the moiré images computed by changing the curvature value from 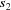 = 0.045′′ mm^−1^ to 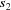 = −0.045′′ mm^−1^ are shown in Figs. 7 ▸(*a*), 7 ▸(*b*); the other numerical conditions for this computation were kept the same as in the case of Figs. 5 ▸(*b*), 5 ▸(*c*), though the image in Fig. 7 ▸(*a*) was obtained with a slightly smaller value of 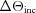 than that for Fig. 5 ▸(*b*), in order to obtain the best image (see figure captions of Figs. 5 ▸ and 7 ▸). In the OE fringes in Fig. 7 ▸(*a*) the fringe slope is observed to occur in the opposite direction to that in Fig. 5 ▸(*b*) for 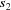 = 0.045′′ mm^−1^. The slope angle was −7.3° from the *x* axis in the direct measurement on the image. In the BH fringes in Fig. 7 ▸(*b*) the wave form of the fringe-line oscillation is observed to lean to the left side, unlike that in the case of Fig. 5 ▸(*c*). From the study in some detail by computation, the lean of the oscillation wave form was always to this direction in the case of 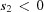, except when 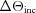 was very small.

### 3.3. Moiré images of bicrystals of *t*_1_ = *t*_2_ = 1.5 (μ*t*_1,2_ = 2.193) and *t*_1_ = *t*_2_ = 1.6 (μ*t*_1,2_ = 2.339)   

**Figure 8:**
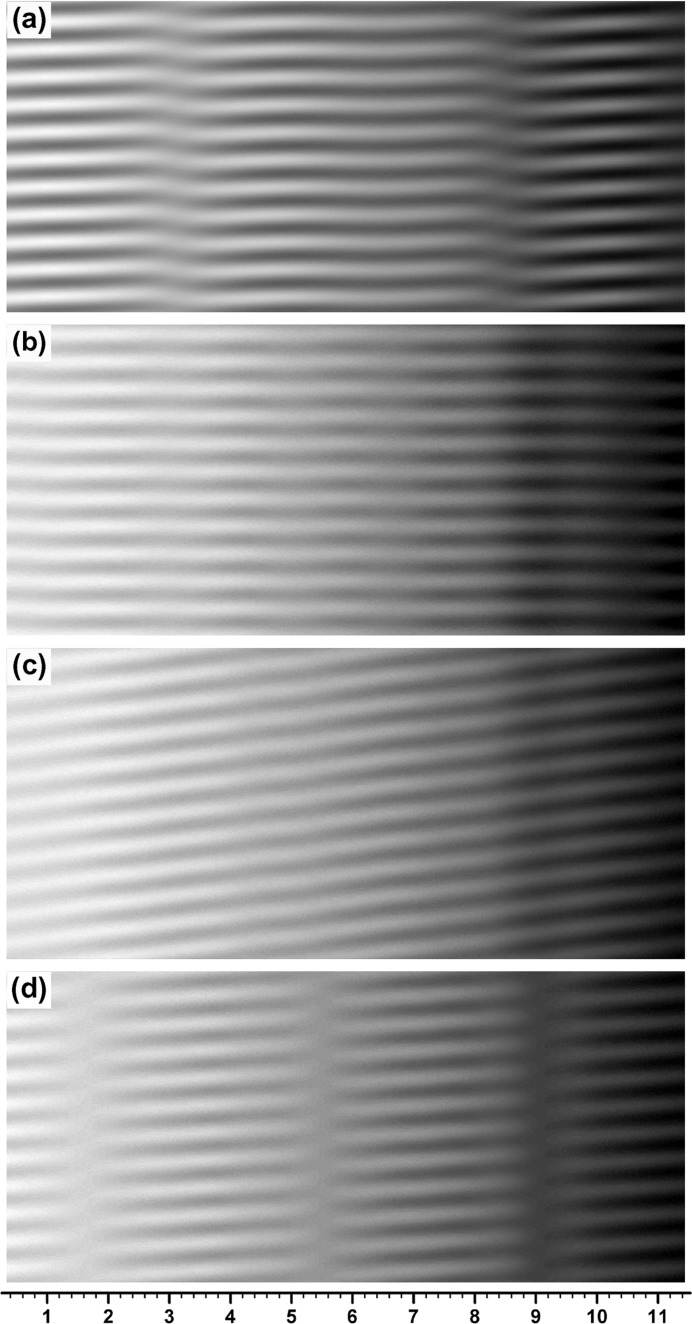
Moiré images computed for bicrystal models of 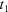 = 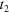 = 1.5 and 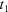 = 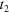 = 1.6. The thicknesses of the component crystals and the incident-wave angular widths for the computed images are as follows: (*a*) 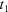 = 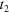 = 1.5, 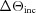 = 0.36′′; (*b*) 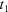 = 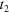 = 1.5, 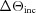 = 0.90′′; (*c*) 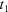 = 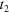 = 1.6, 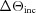 = 0.90′′; (*d*) 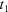 = 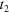 = 1.6, 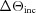 = 1.50′′. Other numerical conditions for the computation are the same as for Fig. 5 ▸.

Examples of moiré images with 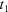 = 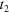 = 1.5 and 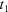 = 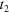 = 1.6 (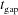 = 0.24) are shown in Figs. 8 ▸(*a*)–8 ▸(*d*). Similar to the case of 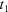 = 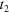 = 0.8 and 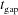 = 0.24 in Section 3.2, the appearance of images in this case when 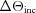 is small (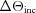 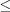 0.12′′) was similar to that of the plane-wave image in Fig. I-10(*a*) where fringe lines show a gentle oscillation. However, the oscillation amplitude of the fringe lines was much smaller in this case than those in the plane-wave image and in the integrated image of 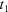 = 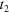 = 0.8, and therefore the fringes practically looked like HS fringes. When the angular width 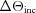 was increased to 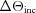 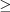 0.16′′, fringe lines came to show a clearly discernible oscillation, with LC bands appearing in the image. As described in Section 3.2, OE and BH fringe patterns appeared roughly alternately with the increase in 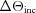, though in this case (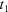 = 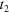 = 1.5) the BH fringe pattern seemed to occur more predominantly than the OE fringe pattern. Fig. 8 ▸(*a*) shows a moiré image of the BH fringe pattern when 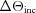 = 0.36′′, as an example in this domain of 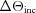. Compared with the image with 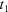 = 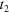 = 0.8 in Fig. 5 ▸(*c*), the step-down (or step-up) of fringe lines at LC band sites (*x* ≃ 3.0, 8.6) becomes smaller, owing to the increase in the crystal thicknesses. Of the images presented in this paper, this image was computed under the nearest condition to the previous experimental moiré images [see Fig. I-1 in Paper I; further see Yoshimura (1996 ▸, 1997 ▸)]. Two LC bands are observed in this image, while three LC bands appeared in the experimental image with nearly the same width in the *x* direction. This disagreement is considered to be due to the fact that the employed values of 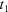, 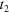 and 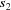 do not exactly agree with the values of the experimental images. When a further moiré image was computed by assuming that 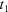 = 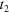 = 1.6 and *s*_2_ = 0.05′′ mm^−1^ (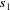 = 0), the number of LC bands in the resulting image agreed approximately with that in the experimental image.

When 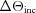 was increased to 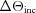 ≃ 0.9′′, the resulting moiré image was the one shown in Fig. 8 ▸(*b*). The moiré image again became very close to the HS fringe pattern, with a fringe-line oscillation of a very small amplitude and an almost disappearing LC band pattern. The fringe pattern did not change significantly from the one in Fig. 8 ▸(*b*) with further increase in 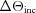, though some exceptional images of low contrast were produced at exceptional values of 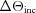 such as 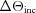 = 1.0′′, 1.5′′ *etc*. With the gap width of 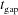 = 0.24 and the crystal thicknesses of 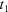 = 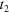 = 1.5, the fringe contrast decreased rapidly with increase in 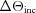, and almost disappeared with 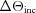 ≃ 1.5′′ (see fringe contrast diagram in Fig. 12).

As has been shown so far, the fringe contrast 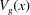 varies in an oscillating way with the *x* coordinate in the image, and also varies in an oscillating way with the angular width 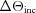; the fringe pattern also varies with 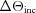 in an oscillating way when 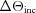 is in the middle-magnitude domain. In addition to such dependences on *x* and 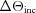, the fringe contrast and the fringe pattern seem to vary in an oscillating way with the crystal thicknesses 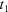 and 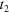, as seen from the results of this trial simulation research. The variations in 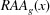, 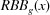 and 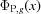 with 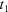 = 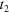 = 1.5 seem to be more gentle than with 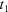 = 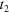 = 1.4 and 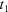 = 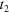 = 1.6. Figs. 8 ▸(*c*) and 8 ▸(*d*) show moiré images with 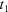 = 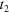 = 1.6 as an example of the case where a clear variation of fringe pattern continues up to a larger value of 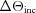 than in the case of 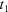 = 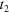 = 1.5. In Fig. 8 ▸(*c*), which is compared with Fig. 8 ▸(*b*) with 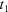 = 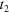 = 1.5, an OE fringe pattern is produced even with 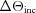 ≃ 0.9′′. The fringe pattern continued to vary with further increase in 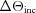, and almost settled to the pattern shown in Fig. 8 ▸(*d*) with 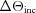 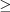 1.5′′. It may be noted that this fringe pattern is similar to that in Fig. 5 ▸(*d*) with 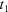 = 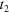 = 0.8, apart from the differences in the position where LC bands occur and in the fringe contrast. The slope angle of the fringes is about 3.5° in this case also.

### 3.4. Moiré images of bicrystals of *t*_1_ = *t*_2_ ≥ 2.5 (μ*t*_1,2_ ≥ 3.655)   

**Figure 9:**
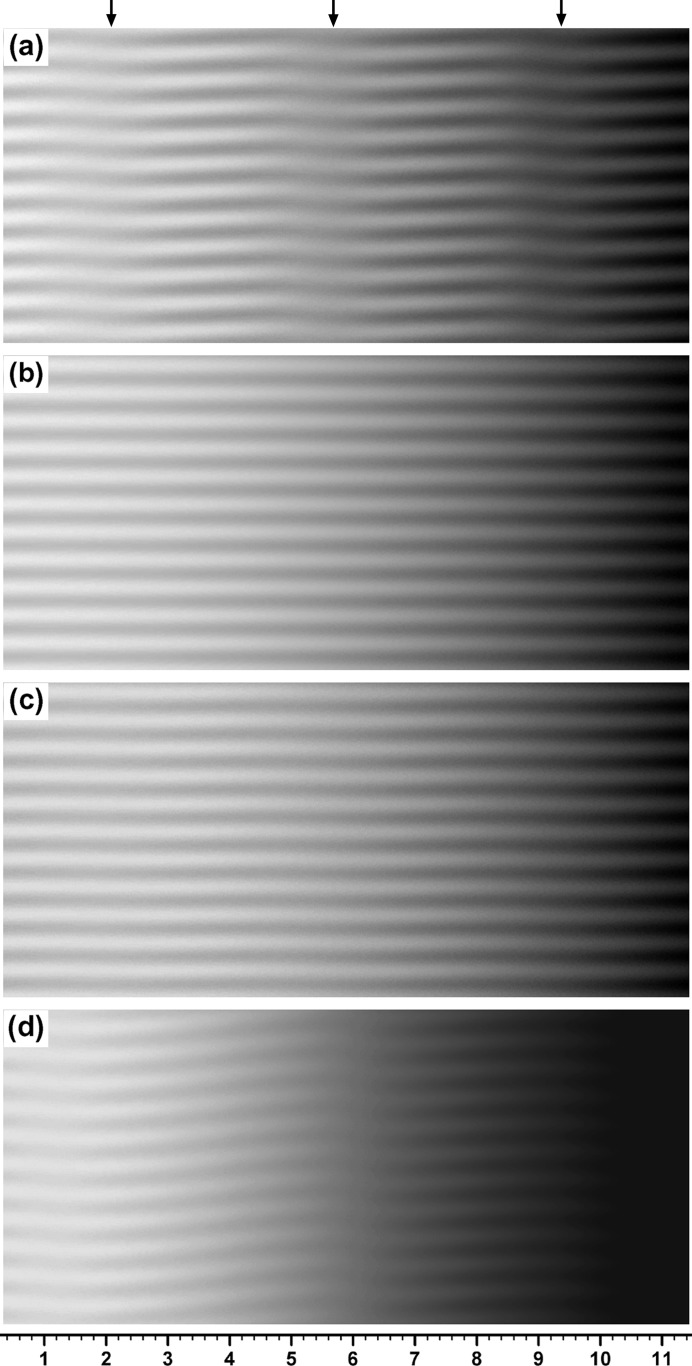
Moiré images computed for bicrystal models of 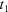 = 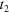 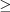 2. The thicknesses of the component crystals and the incident-wave angular width for the computed images are as follows: (*a*) 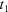 = 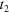 = 2.0, 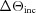 = 1.08′′; (*b*) 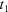 = 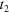 = 2.5, 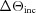 = 0.90′′; (*c*) 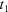 = 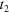 = 3.0, 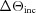 = 0.90′′; (*d*) 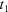 = 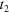 = 3.0, 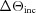 = 1.0′′. Other numerical conditions for the computation are the same as for Fig. 5 ▸.

**Figure 10:**
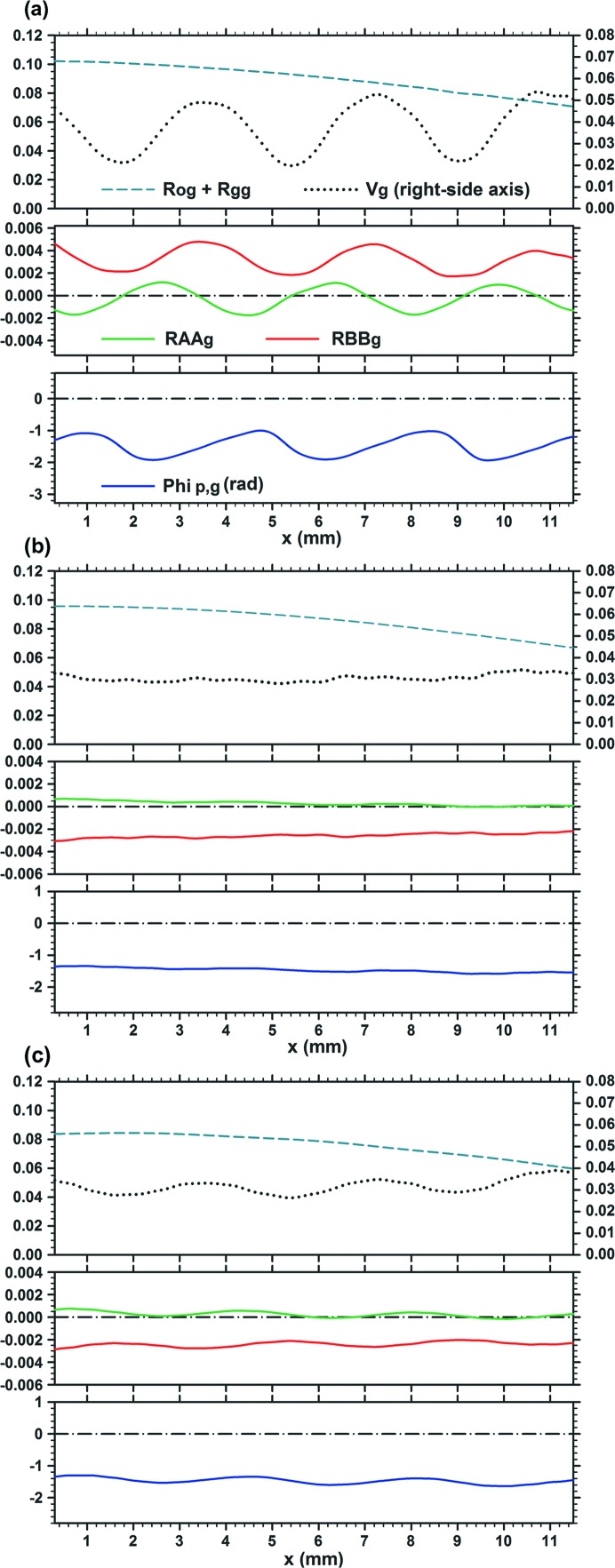
Characteristic curves of the moiré images in Fig. 9 ▸. Graphs (*a*), (*b*) and (*c*) are related to the moiré images (*a*), (*b*) and (*c*), respectively, in Fig. 9 ▸. For further information see text.

When the crystal thicknesses were increased from 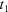 = 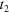 = 1.5 and 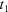 = 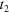 = 1.6, the fringe patterns that were produced and their variations with 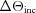 were basically analogous to the results for 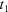 = 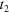 = 0.8, 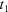 = 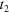 = 1.5 and 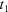 = 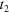 = 1.6, until the thickness was increased to 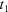 = 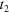 ≃ 2.5. Figs. 9 ▸ and 10 ▸ show examples of moiré images with 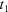 = 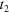 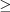 2.5 and graphs of the characteristic curves concerned. Figs. 9 ▸(*a*) and 10(*a*) show, respectively, a moiré image and the characteristic curves with 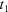 = 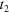 = 2.0, to be compared with those with 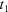 = 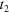 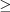 2.5. The LC bands and the fringe-line bends are still observed at the positions *x* ≃ 1.8, 5.4, 9.0 (indicated by arrows), though the fall in fringe contrast is considerably moderated. When the crystal thicknesses were increased further, a similar aspect of the produced fringe patterns continued up to 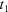 = 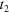 = 2.4. However, with 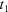 = 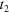 ≃ 2.5, the OE fringe patterns and BH fringe patterns with LC bands disappeared almost completely, and all the fringe patterns became of HS fringes as shown in Fig. 9 ▸(*b*), except for some exceptional cases. From the graph of the characteristic curves in Fig. 10 ▸(*b*), it is seen that this transition to HS fringes results from the convergence of 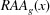 and 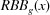 to their respective finite values, unlike the case of Figs. 1 ▸(*c*) and 2 ▸(*c*) with 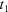 = 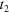 = 0.8 and 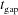 = 0.

When the crystal thicknesses became 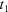 = 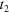 = 2.6, OE and BH fringe patterns appeared again, though with weak LC bands, for the reverse tendency in the dependence on the crystal thicknesses as shown in Figs. 8 ▸(*c*) and 8 ▸(*d*). However, when the crystal thicknesses were further increased, the fringe patterns produced again became of HS fringes as seen when 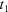 = 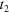 = 2.5. For the crystal thicknesses approximately 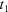 = 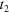 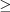 3, the fringe patterns produced were always nearly of HS fringe type, in spite of the increase in 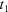 and 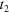. This result indicates that the minimum specimen thickness for obtaining a good moiré image without disturbance from PL oscillation and crystal strain is 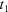 = 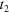 ≃ 3 (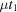 = 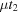 ≃ 4.4). Figs. 9 ▸(*c*) and 10 ▸(*c*) show, respectively, a moiré image with 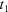 = 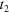 ≃ 3 and a graph of the characteristic curves concerned. The values of the angular width 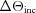, with which the produced fringe patterns were not of HS fringes, were 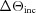 = 0.5′′, 1.0′′, 1.5′′ *etc*. with any value of 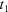 or 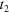. With these values of 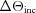, the image intensity and fringe contrast momentarily fell markedly, and OE or BH fringe patterns appeared in a disordered manner in a low-intensity image. Fig. 9 ▸(*d*) shows an example of such a specific moiré image with 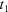 = 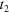 = 3.0 and 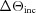 = 1.0′′. The reason for this specificity is as yet unknown.

### 3.5. Diffraction intensity curves for the presented moiré images and wide-view diagram of the fringe contrast variation   

#### 3.5.1. Curves of mean image intensity versus Δθ_m_   

**Figure 11:**
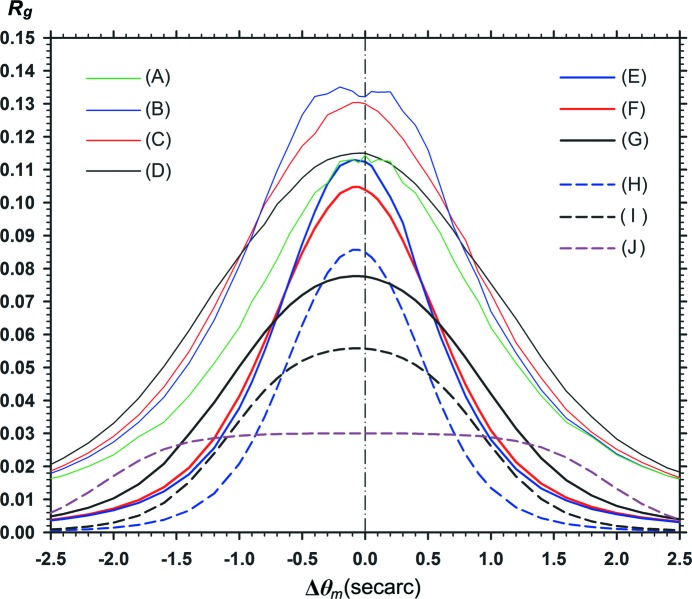
Plots of the mean intensity of moiré images [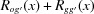] versus the mid deviation angle 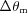. Numerical conditions for the computation are as follows: curve (*A*) 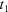 = 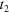 = 0.8, 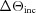 = 0.5′′, 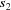 = 0; (*B*) 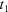 = 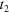 = 0.8, 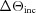 = 0.5′′; (*C*) 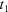 = 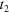 = 0.8, 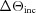 = 1.2′′; (*D*) 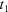 = 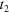 = 0.8, 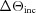 = 2.0′′; (*E*) 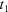 = 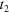 = 1.5, 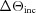 = 0.36′′; (*F*) 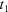 = 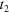 = 1.5, 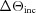 = 0.9′′; (*G*) 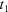 = 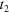 = 1.5, 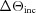 = 2.0′′; (*H*) 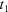 = 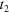 = 2.5, 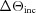 = 0.9′′; (*I*) 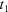 = 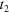 = 2.5, 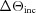 = 2.0′′; (*J*) 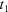 = 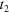 = 2.5, 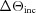 = 4.0′′. The curvature strength of the rear crystal was *s*_2_ = 0.045′′ mm^−1^ for all the curves from (*B*) to (*J*). The half widths (FWHM) of these intensity curves are: 2.18′′, 2.15′′, 2.55′′ and 2.79′′ in curves (*A*), (*B*), (*C*) and (*D*), respectively; 1.42′′, 1.57′′, 2.27′′ in curves (*E*), (*F*) and (*G*), respectively; 1.06′′, 2.09′′, 4.01′′ in curves (*H*), (*I*) and (*J*), respectively.

Fig. 11 ▸ gives diffraction intensity curves that show the dependence of the mean image intensity of moiré images on the mid deviation angle 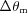, for the moiré images presented in this paper so far. The mean image intensity 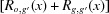 averaged over the represented range of moiré images 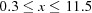 is plotted. The value of intensity is represented as the ratio of the integrated image intensity to the total incident intensity 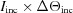 [see equations (12*a*)[Disp-formula fd12a], (12*b*)[Disp-formula fd12b]]. These intensity curves would approximate well real rocking curves measured experimentally. The curves (*B*)–(*H*) are related to the presented moiré images as follows: (*B*) Figs. 1 ▸(*b*) and 3 ▸(*a*), (*C*) Fig. 5 ▸(*c*), (*E*) Fig. 8 ▸(*a*), (*F*) Fig. 8 ▸(*b*) and (*H*) Fig. 9 ▸(*b*). These intensity curves are the same for all values of 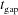, including the case of 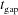 = 0. This is because the factor 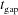 is not involved in the functions 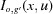 and 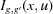 which are the integrands in 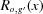 and 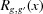 [see equations (I-22*a*,*b*)]. Furthermore, the intensity curves do not depend on the value of 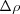, which gives the spacing of moiré fringes (in the case of rotation moiré), and are the same for all values of 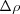. The reason for this is also that the factor 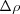 is not involved in 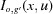 and 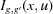.

The curve (*A*) is the intensity curve when the curvature deformation is not induced, *i.e.* 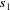 = 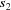 = 0, with 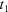 = 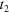 = 0.8. Unlike the other curves (*B*)–(*J*), this curve has a symmetric shape with respect to the coordinate origin 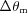 = 0. This characteristic is understood as follows: though the integrand functions 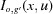 and 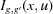 in 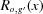 and 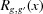 are both asymmetric functions, the function 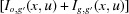 becomes symmetric with respect to *u*, *i.e.* 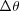 (*u* = 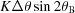), since the relationship 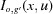 = 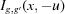 holds when 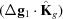 = 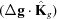 = 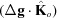 = 0 [*i.e.* 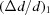 = 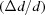 = 0, 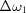 = 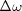 = 0 (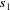 = *s* = 0)] and 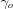 = 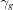, 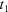 = 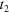. When these conditions do not hold, even in part, the function 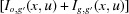 becomes asymmetric. Based on this understanding, it is considered to be due to the failure of the condition *s* = 0 that the curves (*B*), (*C*) *etc*. are asymmetric as a whole, with their peak positions slightly shifted to the low-angle side. In fact, when the computation was performed on the assumption that 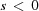, or when the *x* position corresponding to the centre of curvature was moved to the left side with 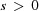, the peak position of the intensity curves was displaced to the high-angle side.

As to the half width (FWHM) of the intensity curves, that of the curve (*A*) was 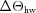 = 2.18′′, while the half width of the intrinsic diffraction curve with no absorption is 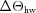 = 2.29′′. The half widths of the other curves were as described in the figure caption. The image intensity decreases fairly rapidly with increase in the crystal thicknesses, due to the increased absorption of X-rays. When the angular width 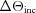 is increased, the peak of the intensity curves is lowered under the present definition of diffracted intensity, while their half widths increase. When 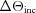 becomes larger than *circa* 2′′, the increase in the half width with 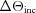 proceeds relatively rapidly. The curve (*J*) (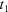 = 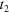 = 2.5) is an example of a curve of a large half width (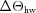 = 4.01′′) with 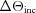 = 4′′. When 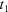 and 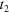 took values other than those in this case, intensity curves with 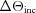 = 4′′ were also of a similar wide flat-top shape but with a slight difference in the top height; this was also the case when 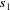 = 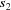 = 0. Such shapes with a wide flat top are rarely seen for X-ray rocking curves. The intensity curve with 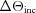 = 10′′, which is related to the moiré images in Figs. 1 ▸(*c*), 3 ▸(*b*) and 5 ▸(*d*), would be like a low-height horizontal line if it is plotted in the same graph of Fig. 11 ▸.

#### 3.5.2. Curves of mean fringe contrast versus ΔΘ_inc_   

**Figure 12:**
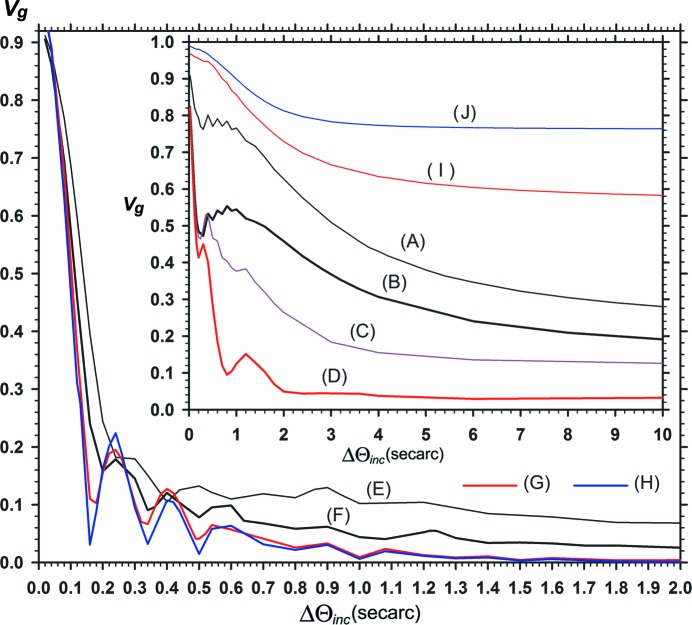
Plots of the mean fringe contrast of computed moiré images versus the incident-wave angular width 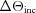. Numerical conditions for the computation are as follows: curve (*A*) 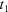 = 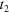 = 0.8, 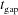 = 0.0, 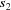 = 0; (*B*) 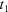 = 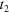 = 0.8, 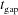 = 0.0; (*C*) 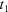 = 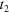 = 0.8, 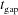 = 0.02; (*D*) 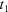 = 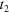 = 0.8, 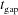 = 0.05; (*E*) 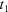 = 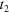 = 0.8, 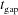 = 0.2; (*F*) 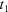 = 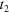 = 0.8, 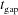 = 0.24; (*G*) 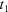 = 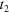 = 1.5, 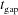 = 0.24; (*H*) 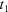 = 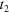 = 2.5, 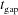 = 0.24; (*I*) 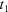 = 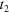 = 1.5, 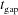 = 0.0; (*J*) 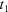 = 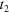 = 2.5, 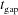 = 0.0. The curvature strength of the rear crystal was *s*_2_ = 0.045′′ mm^−1^ for all the curves from (*B*) to (*J*).

Fig. 12 ▸ shows the characteristic curves of the fringe contrast variation with the angular width 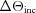. Similar to the image intensity curves in Fig. 11 ▸, fringe contrasts 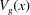 averaged over the represented range of moiré images 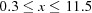 are plotted. The curve (*A*) in the inset shows the variation of the fringe contrast related to the moiré image when 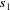 = 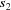 = 0 with 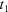 = 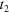 = 0.8 and 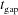 = 0. The other curves (*B*), (*D*) *etc*. are related to the moiré images presented so far as follows: (*B*) Fig. 1 ▸, (*D*) Fig. 3 ▸, (*F*) Fig. 5 ▸, (*G*) Figs. 8 ▸(*a*), 8 ▸(*b*) and (*H*) Fig. 9 ▸(*b*) [*s*_2_ = 0.045′′ mm^−1^ in all the curves of (*B*) to (*J*)]. These fringe contrast curves change with the curvature values 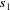 and 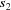, but do not change with the value of 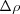. The reason is that 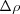 is not involved in the expression of 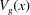 in equation (15). While it has been observed in Figs. 2 ▸, 4 ▸, 6 ▸ *etc*. that the fringe contrast oscillates with the *x* coordinate in the image, the oscillation with the 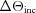 value in the mean fringe contrast 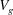 is seen understandably in this graph. The 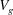 oscillation occurs in the domain of relatively small 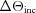, and nearly converges in the domain of 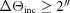 (though a very weak oscillation continues further). The curves of 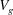 variation gradually descend with 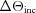, and almost settle to the given values. Like the 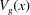 oscillation with the *x* coordinate, the oscillation with 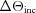 in the mean fringe contrast 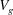 would also be related to the oscillations in 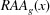 and 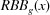, which arise from the combined effect of the PL oscillation and gap phase difference.

With the increase in the crystal thicknesses 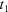, 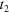 and the gap width 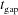, the curves of 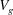 variation descend rapidly with increase in 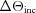 [curves (*D*)–(*H*)]. In the domain of small 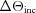, the 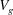 curves oscillate with a large amplitude for a small change in 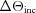. In the case of 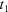 = 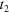 = 2.5 [curve (*H*)], changes in the fringe contrast corresponding to the minima and maxima of the 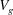 curve were recognized clearly among the moiré images, although it was not so clear in the case of 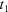 = 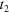 = 1.5 [curve (*G*)]. It must be added that no other co-occurring change was found in these moiré images, in spite of the large, rapid change in the fringe contrast. By adjusting the magnitude of 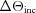, it would be possible to adjust the fringe contrast in the obtained moiré images to a fair extent. This would be useful knowledge when planning future moiré experiments.

To expand on the case of low fringe contrast, the moiré fringe pattern can be fairly well observed with the contrast 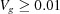, so far as this computer-simulation study (256 graduations) is concerned, aside from the answer given by the experiment. However, with 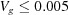 [at 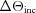 ≃ 1.0′′, 1.5′′ on the curves (*G*) and (*H*)] the fringe pattern became almost invisible even in this simulation study. In the following we assume that 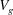 = 0.01 is the visibility limit of the fringe pattern. According to this graph in Fig. 12 ▸, the fringe contrast becomes practically zero with 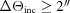 in the integrated images with 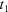 = 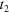 = 1.5 and 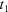 = 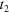 = 2.5 (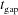 = 0.24). This gives an accurate theoretical explanation for the known experimental fact that the moiré image from a bicrystal specimen (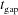 ≃ 0.25) which appears for the quasi-plane-wave incidence (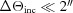) becomes invisible in Lang traverse topography (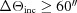) (though visible in section topography). Furthermore, the graph in Fig. 12 ▸ suggests that the moiré image could be observed with some fringe contrast even by Lang topography, if the sample crystal was somewhat thinner than that mentioned. In the results of the computation, the fringe contrast was 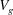 = 0.015 with 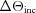 = 60′′, when 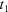 = 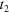 = 0.8 and 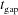 = 0.24 [curve (*F*)]. Additionally, when the gap width was increased with the crystal thicknesses kept at 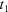 = 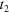 = 1.5, the fringe contrasts obtained by the computation were 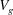 = 0.042 for 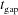 = 0.5, 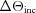 = 0.5′′, 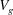 = 0.026 for 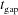 = 0.5, 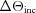 = 1.0′′, and 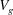 = 0.021 for 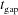 = 1.0, 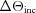 = 0.5′′. These estimates suggest that moiré fringes can be observed even with large gap widths if the angular width of the incident wave is suitably narrowed.

Curves (*I*) and (*J*) (in the inset) show the 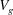 variation curves with 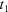 = 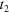 = 1.5, 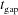 = 0 and with 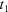 = 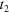 = 2.5, 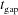 = 0 for reference. The curves (*G*) and (*H*), which give a low fringe contrast with 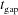 = 0.24, change to such curves as above, giving a high fringe contrast with 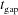 = 0. In these cases, while the image intensity [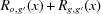] is decreased considerably, the fringe contrast 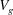 is increased greatly.

## 4. Concluding remarks   

(i) As described above, in connection with the question of what type of moiré images are given by the X-ray moiré-fringe theory in Paper I when practical experimental conditions are applied, we have simulation-computed and observed many integrated moiré images for an assumed bicrystal specimen; this was done by changing the crystal thicknesses and incident-wave angular width over a wide range, and by setting the width of the interspacing gap to different values. It was shown that the interference pattern of intrinsic moiré images is considerably modified by the combined effect of PL oscillation and gap phase difference, related to the effect of strain in the specimen. As examples of such modified and peculiar fringe patterns in the integrated images, the OE fringe pattern and BH fringe pattern were observed. While the BH fringe pattern has been observed in previous experiments, occurrence of the OE fringe pattern in this study is quite a new finding. The experimental verification of it is hoped to be performed in the near future. Furthermore, not only the images of this newly found fringe pattern, but also all the moiré images shown by the present computations are hoped to be really observed and verified in future experiments, although there is no particular question on the correctness of the computed images..

(ii) This theory of moiré fringes was developed by assuming a gapped bicrystal as the specimen for moiré images. It is related to previous experimental studies by the author (*e.g*. Yoshimura, 1996 ▸, 1997 ▸), where gapped bicrystals were used as the specimen. However, studies of X-ray moiré fringes are made mainly using an X-ray interferometer at present, and therefore this theory of moiré fringes should be extended so as to be applicable for interferometer moiré fringes. Nevertheless, the description in this paper has been made with the intention of understanding fully the properties of bicrystal moiré fringes. As a result of such moiré-fringe study, the relationship of fringe contrast versus angular width of the incident wave was extensively studied as shown in the graph in Fig. 12 ▸. As a discussion of this graph, the lower limits of the visibility of bicrystal moiré fringes were described, and it was suggested that if the angular width of the incident wave is narrowed sufficiently, moiré fringes could be observed even with a gap width of 0.5, 1.0 or more (mm). This result would be worthy of attention although its validity must be further studied by experiment. To the best of the author’s knowledge, this theory of moiré fringes would be the first theory applicable to the exact and detailed theoretical understanding of moiré images observed in experiments. Though the topic of X-ray moiré fringes does not attract much interest at present, the theory could be useful for future studies of diffraction moiré fringes, and for the development of related techniques.
